# Rapunzel Syndrome: A Rare Case of Small Bowel Intussusception in a Child

**DOI:** 10.7759/cureus.17911

**Published:** 2021-09-12

**Authors:** William T Head, Raphael H Parrado, Lucas McDuffie

**Affiliations:** 1 Surgery, Medical University of South Carolina, Charleston, USA; 2 Pediatric Surgery, Medical University of South Carolina, Charleston, USA

**Keywords:** bezoar, hair, abdominal pain, small bowel intussusception, gastrointestinal obstruction

## Abstract

Trichobezoars are indigestible masses of ingested hair commonly found in the stomach, often presenting with symptoms related to gastric outlet obstruction and severity related to the mass’s size and location. Gastrointestinal complications include ulceration, perforation, peritonitis, pancreatitis, obstructive jaundice, pneumatosis intestinalis, and intussusception. Management of trichobezoars differs from that of other forms of bezoars, which can often be addressed with chemical dissolution. Trichobezoars are high-density structures that are also resistant to enzymatic and pharmacotherapy degradation, and as such, they require endoscopic, or more commonly, surgical removal. Here, we present the diagnosis and surgical management of a 12-year-old female with a large trichobezoar causing gastric outlet obstruction, with an associated Rapunzel syndrome manifesting as multiple small intestinal intussusceptions.

## Introduction

A *bezoar* is an indigestible mass that accumulates in the gastrointestinal tract after intentional or accidental ingestion of diverse materials such as vegetable matter (phytobezoars - 40%), medications (pharmacobezoars), or hair (trichobezoars) [1]. The location of the impaction varies as well, with the majority in the stomach, followed by the duodenum and the esophagus [2]. Patients may be asymptomatic or present with a spectrum of gastrointestinal symptoms, ranging from mild symptoms such as pain, nausea, and/or anorexia to more severe symptoms such as perforation or obstruction from mass effect or intussusception [2-8]. Early diagnosis and treatment of bezoars are therefore vital, with the management approach largely dependent on the type of material ingested. Amongst bezoars at large, trichobezoars are unique; hair is resistant to enzymatic digestion, resulting in impaction within the gastric body with the potential for extension through the pylorus and into the small bowel, termed Rapunzel syndrome [1,9-12]. We describe an adolescent that presented with progressive obstruction from a trichobezoar with associated Rapunzel syndrome.

## Case presentation

**Figure 1:**
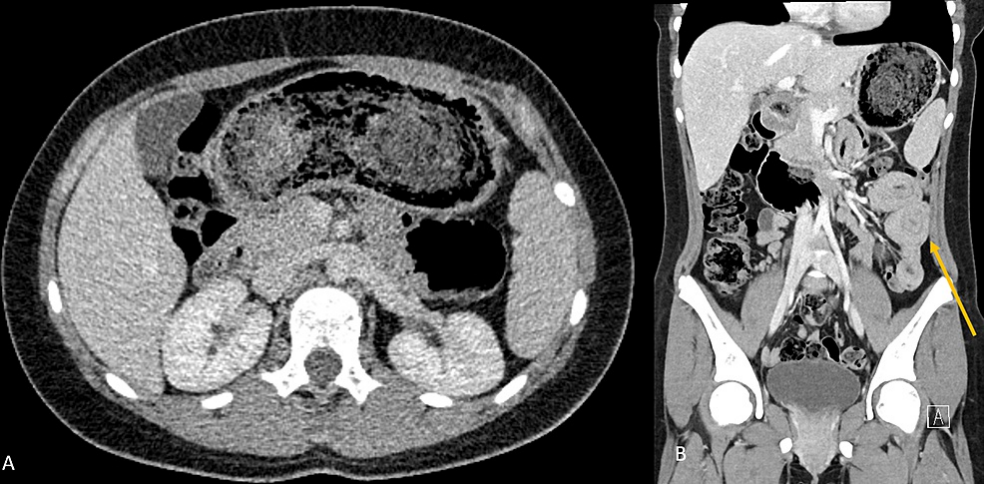
Computerized tomography. Coronal (A) view showing heterogeneous mass in the stomach consistent with a bezoar. Sagittal (B) view with evidence of small bowel to small bowel intussusception (yellow arrow) as well as a heterogeneous mass in the stomach.

A 12-year-old female with no significant past medical or psychiatric history presented with sharp, crampy abdominal pain of three weeks duration associated with nausea and non-bloody, non-bilious emesis. Physical exam was notable for mild tachycardia and hypertension, abdominal tenderness, and voluntary guarding in the right upper and lower quadrants without peritonitis. No hematologic or metabolic derangements were noted on initial labs. Initial abdominal radiographs and a right upper quadrant ultrasound performed at our institution similarly demonstrated no acute abnormalities. Computerized tomography revealed a large gastric bezoar distending the stomach, likely resulting in partial gastric outlet obstruction. Multiple prominent short segments small bowel-small bowel intussusceptions were also noted (Figures 1A, 1B). The decision was made to take her to the OR for exploration and removal of the bezoar.

**Figure 2:**
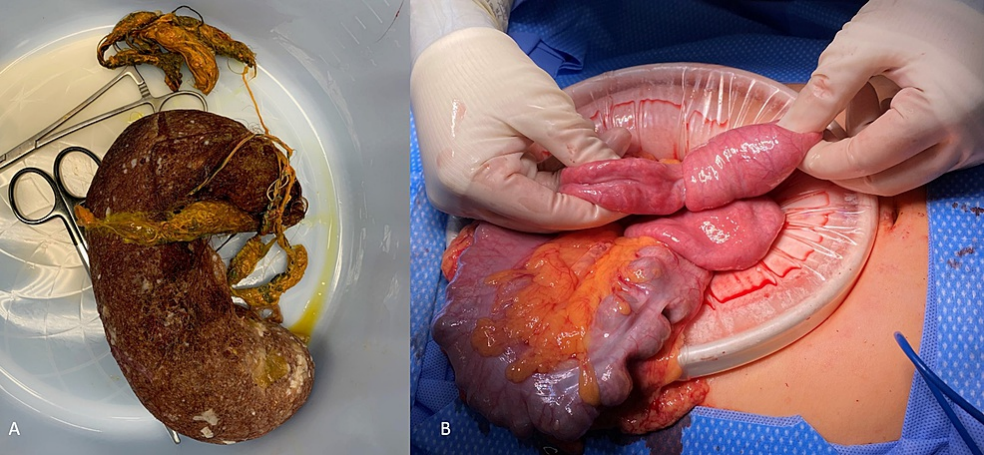
Intraoperative photos. (A) Large bezoar in the shape of the stomach with extension past the pylorus into the small bowel. (B) Small bowel to small bowel intussusception caused by the extracted bezoar.

An upper midline laparotomy with an oblique gastrotomy was performed. Intraoperatively, a large gastric trichobezoar was found with extension past the ligament of Treitz into the mid-jejunum. A long braided synthetic cord measuring about 50 cm was also present and intertwined with the hair, serving as a lead point for multiple small bowel-small bowel intussusceptions (Figures 2A, 2B). The trichobezoar was removed, and the gastrotomy site was closed in two layers. The intestines were then examined sequentially, which led to the discovery of seven proximal intussusceptions that were each manually reduced with an appreciation of viable tissue along the corresponding intestinal tract. No complications occurred, with minimal estimated blood loss.

On further questioning, the patient and her parents endorsed hair chewing throughout her life, but denied overt hair pulling or ingestion. She was also found to have stressors concerning schoolwork with several prior episodes of self-harm behavior. Ultimately, she was diagnosed with a social anxiety disorder with trichophagia and was referred for outpatient psychological therapy to further evaluate possible trichotillomania. The remainder of her hospital course was unremarkable, and she was discharged on postoperative day 3.

## Discussion

Bezoars in and of themselves are not dangerous, yet the potential for obstruction of the gastrointestinal tract warrants urgent diagnosis and treatment. We present a case of a trichobezoar with Rapunzel syndrome leading to seven small bowel-small bowel intussusceptions. Trichobezoars are distinct from other bezoars in that hair can generate highly dense masses that are resistant to enzymatic digestion. Furthermore, the smooth surface is thought to prevent hair from passing with peristaltic waves and to be retained within the gastric folds [3,13,14]. The extensive length of the bezoar inevitably served as a lead point for the development of intussusceptions further along the small intestine. While others have noted that trichobezoars can separate into smaller parts and cause obstruction at several levels of the gastrointestinal tract, this case highlights the potential for ischemia and necrosis at various levels due to concurrent intussusceptions from a single, contiguous object causing multiple lead points along its length [15-17].

The diagnosis of trichobezoars is often delayed, as they are not often recognized on initial presentation due to non-specific abdominal symptoms [13]. The initial use of plain radiographs and sonography may hinder the diagnosis further. Reports have shown that 20%-60% of bezoars can be identified by plain film and ultrasound [18]. The density of hair makes identification difficult, yet gastric distension and obstruction should be readily visible using these two methods. Computerized tomography is able to identify 97% of bezoars in addition to determining the location, cause, and degree of obstruction and other relevant sequelae. Our case further highlights the value of computerized tomography and demonstrates that bezoars should be included on the differential in patients with recurrent, nonspecific abdominal symptoms and atypical eating habits.

Rapunzel Syndrome was first described by Vaughan et al. in 1968 and has since been reported with varying features [19]. A review of these cases identified three unifying criteria: (1) a trichobezoar with a tail, (2) extension of the tail at least to the jejunum, and (3) symptoms of obstruction [10]. We support these criteria and believe they appropriately represent the pathophysiologic processes found in our case. Prior reviews have suggested that it is a rare sequela of trichobezoars, but a recent case series diagnosed Rapunzel syndrome in 71% of its trichobezoar patients [11].

The treatment of bezoars largely falls into three categories: medical dissolution, endoscopy, and surgery [1]. Trichobezoars are typically resistant to medical dissolution [3]. Endoscopic removal has been used in several isolated cases but is often not successful, particularly for those cases with larger masses. A review by Gorter et al. identified 40 cases in which endoscopic removal was attempted with only two proving successful (5%) [13]. Attempts at endoscopic removal are often prolonged, delaying effective treatment. Up-front laparotomy should be considered the gold standard for large trichobezoars, especially if there is preoperative evidence for Rapunzel syndrome. The decision to pursue enterotomy and/or gastrotomy varies on a case-by-case basis and is largely based on the location, length, overall size, and intestinal viability. Post-operative psychiatric consultation is vital to prevent recurrence in those patients with trichophagia [20].

## Conclusions

Trichobezoars are indigestible masses of ingested hair that can cause gastric outlet obstruction and even in our case intussusception as they serve as a lead point. They can be complicated with ulceration, perforation, pancreatitis, obstructive jaundice, and pneumatosis intestinalis. For this reason, early diagnosis and treatment are vital. They are usually resistant to enzymatic degradation and endoscopic treatment for which laparotomy should be always considered. Our case demonstrates the safety and efficacy of an up-front surgical approach with subsequent psychiatric evaluation and treatment in the management of trichobezoars with associated Rapunzel syndrome.
